# The prognostic effect of mechanical, ultrastructural, and ECM signatures in glioblastoma core and rim

**DOI:** 10.1063/5.0203570

**Published:** 2024-06-24

**Authors:** Bradley J. Mahaffey, Zachary P. Fowler, Zoe Lung, Vivien Dang, Hyunchul Lee, Allison McKenzie Johnson, Marco A. Munoz, Dylan A. Goodin, Hermann B. Frieboes, Brian J. Williams, Joseph Chen

**Affiliations:** 1 Department of Bioengineering, University of Louisville, Louisville, Kentucky 40292, USA; 2 Department of Neurosurgery, University of Louisville, Louisville, Kentucky 40202, USA; 3 Department of Pharmacology and Toxicology, University of Louisville, Louisville, Kentucky 40202, USA; 4 UofL Health—Brown Cancer Center, University of Louisville, Louisville, Kentucky 40202, USA; 5 Center for Predictive Medicine, University of Louisville, Louisville, Kentucky 40222, USA

## Abstract

Glioblastoma (GBM) is a highly invasive, aggressive brain cancer that carries a median survival of 15 months and is resistant to standard therapeutics. Recent studies have demonstrated that intratumoral heterogeneity plays a critical role in promoting resistance by mediating tumor adaptation through microenvironmental cues. GBM can be separated into two distinct regions—a core and a rim, which are thought to drive specific aspects of tumor evolution. These differences in tumor progression are regulated by the diverse biomolecular and biophysical signals in these regions, but the acellular biophysical characteristics remain poorly described. This study investigates the mechanical and ultrastructural characteristics of the tumor extracellular matrix (ECM) in patient-matched GBM core and rim tissues. Seven patient-matched tumor core and rim samples and one non-neoplastic control were analyzed using atomic force microscopy, scanning electron microscopy, and immunofluorescence imaging to quantify mechanical, ultrastructural, and ECM composition changes. The results reveal significant differences in biophysical parameters between GBM core, rim, and non-neoplastic tissues. The GBM core is stiffer, denser, and is rich in ECM proteins hyaluronic acid and tenascin-C when compared to tumor rim and non-neoplastic tissues. These alterations are intimately related and have prognostic effect with stiff, dense tissue correlating with longer progression-free survival. These findings reveal new insights into the spatial heterogeneity of biophysical parameters in the GBM tumor microenvironment and identify a set of characteristics that may correlate with patient prognosis. In the long term, these characteristics may aid in the development of strategies to combat therapeutic resistance.

## INTRODUCTION

Glioblastoma (GBM) is a highly invasive and aggressive brain cancer, carrying a median survival of 10–14 months and a three-year survival rate of less than 5%.^1,2^ These poor prognoses are due to its resistance to standard therapeutic intervention, involving maximal safe resection, chemotherapy, and radiation.^3–5^ The ability of GBM to evade treatment is largely attributed to the heterogeneous tumor microenvironment (TME), which provides biomolecular and biophysical cues that regulate GBM adaptation.^6–8^ Thus, intense effort has been placed on dissecting these interactions to identify TME targeting therapies that can combat tumor plasticity and improve prognosis.

To study the heterogenous TME, studies have focused on two major tumor regions– the core and the rim.^8–10^ The core is dense and hypoxic, while the rim is thought to have higher microvascular density and be more permissive to cell spread and infiltration.^11^ The core and rim microenvironments have distinct biomolecular inputs including metabolites, cytokines, and oxygenation, which facilitate the unique behaviors of each region.^8,12–14^ Although the biomolecular components are critical for GBM progression, emerging studies highlight the dramatic impact of biophysical cues in this milieu.^15–17^ Notably, biophysical inputs such as increased substrate stiffness is sufficient to promote GBM progression and aggressiveness independent of any biomolecular stimulus.^18,19^ However, the biophysical characteristics of the GBM core and rim are largely unexplored.

Biophysical changes in the TME include features such as increased tissue rigidity, aberrant ECM composition, and denser ultrastructure.^15,20^ Each of these alterations can influence GBM progression.^16,21^ For example, increased mechanical rigidity in the TME regulates glioma cell morphology and cytoskeletal organization, leading to more malignant cells as evidenced by increased motility and proliferation.^18^ Also, this effect is maintained in the cancer stem cell population, with elevated substrate stiffness increasing invasive potential.^22,23^ Altered hyaluronic acid (HA) content, a major ECM protein in the brain, has also been associated with CD44-dependent migration and spread, which has been observed in aggressive mesenchymal GBM.^24–27^ Furthermore, changes in tissue density can regulate invasion potential, with denser matrix limiting infiltration of GBM cells.^28^ Collectively, these studies have shown that biophysical parameters are significant regulators of cell phenotype and function; however, the precise alterations present in the GBM TME have yet to be fully characterized.

In this study, we quantified for the first time the biophysical characteristics of human patient-matched GBM core and rim. We find that although there are differences between patients, the biophysical trends are maintained across our patient population. Using atomic force microscopy, we find that the tumor core is stiffer than the rim and non-neoplastic control. Through scanning electron microscopy analysis, we determined that the tumor core has decreased porosity and decreased pore density. Through immunohistochemistry, we find that the tumor core has dramatically increased HA and tenascin-C content. Finally, using patient progression-free survival, we show that these biophysical parameters have prognostic significance. These findings present novel, quantitative insights toward the biophysical alterations that arise in the GBM TME and provide a foundation for deeper interrogation of the interplay between the biophysical GBM TME and GBM cells.

## RESULTS

### Patient demographics

Our patient cohort consisted of seven GBM patients ranging from ages of 59 to 76 years. There were four males and three females, and eight were non-Hispanic White, while 1 was Hispanic or Latino and Black. One non-neoplastic sample was acquired from an epileptic patient who was a 54-year-old non-Hispanic, White female (Table I).

**TABLE I.** Patient demographics and survival data.

	Patient Nos.	Histology	Grade	PFS	Age	Sex	Primary ethnicity	Primary race	OS
GBM	1625	Glioblastoma, IDH1-R132H negative	4	491	62	Female	Non-Hispanic	White	926
	1681	Glioblastoma, IDH1-R132H negative	4	61	73	Male	Non-Hispanic	White	636
	1686	High grade glioma	4	252	74	Male	Non-Hispanic	White	488
	1706	Glioblastoma, IDH1-R132H negative	4	146	76	Female	Non-Hispanic	White	275
	1723	Glioblastoma, IDH negative	4	167	62	Male	Non-Hispanic	White	235
	1867	Glioblastoma, IDH1-R132H negative	4	74	62	Female	Non-Hispanic	White	74
	2005	Glioblastoma	4	143	59	Male	Hispanic or Latino	Black	169
CTL	1943	Intractable epilepsy	X	495	54	Female	Non-Hispanic	White	495

### Glioblastoma core is mechanically stiffer than the rim

**FIG. 1.**
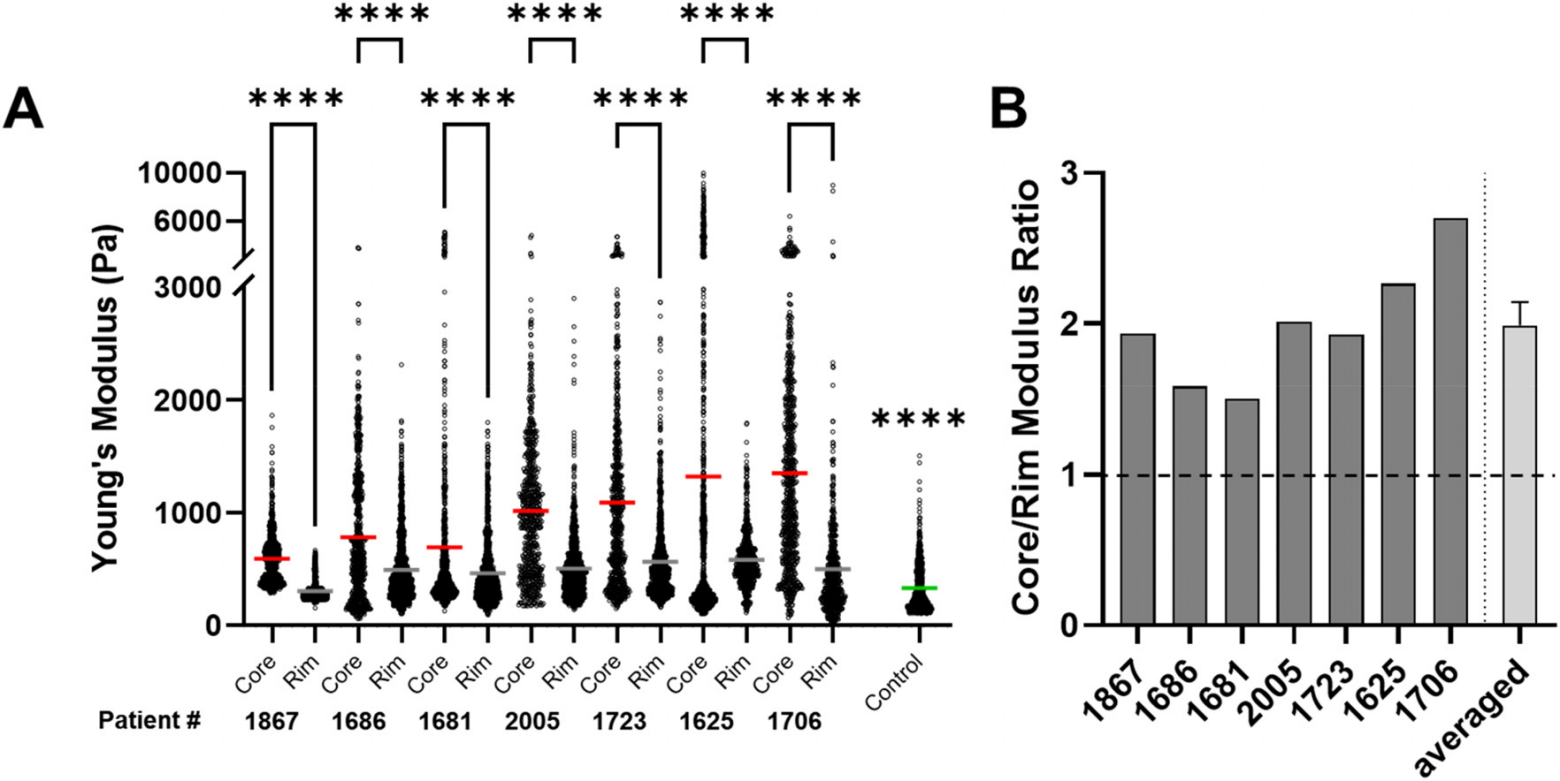
AFM reveals spatial heterogeneity and stiffer core tissue. GBM tumor exhibits heterogeneous mechanical signatures with tumor core exhibiting higher stiffness when compared to tumor rim and non-neoplastic controls. Significant changes were observed with each core and rim pair, and the control was significantly different from all except 1867 rim. A minimum of 1280 force curves were processed for each patient across three tumor regions (a). The relative differences between tumor core and rim is patient-specific; however, the core is stiffer than the rim across patients (b). Statistical significance was determined via one-way ANOVA and Tukey's multiple comparisons test; ^****^ denotes p < 0.0001.

Using AFM, we measured the mechanical properties of patient-matched core and rim tissues and non-neoplastic control tissue. We found that these regions were dramatically different, with tumor cores being universally stiffer than the rim, revealing a spatial heterogeneity in tissue mechanics across the same tumor [Fig. 1(a)]. Tumor core stiffnesses ranged from 600 Pa to 1.3 kPa, while rim tissue ranged from 300 to 500 Pa, and non-neoplastic tissue measured at 330 Pa. Furthermore, although the magnitude of core and rim stiffnesses differed across patients, a consistent trend of core/rim ratio was observed with all patients exhibiting > 1.5 core/rim ratio. An average core/rim modulus ratio of 1.98 was determined across the patient cohort [Fig. 1(b)].

### Glioblastoma core has decreased porosity and pore density

**FIG. 2.**
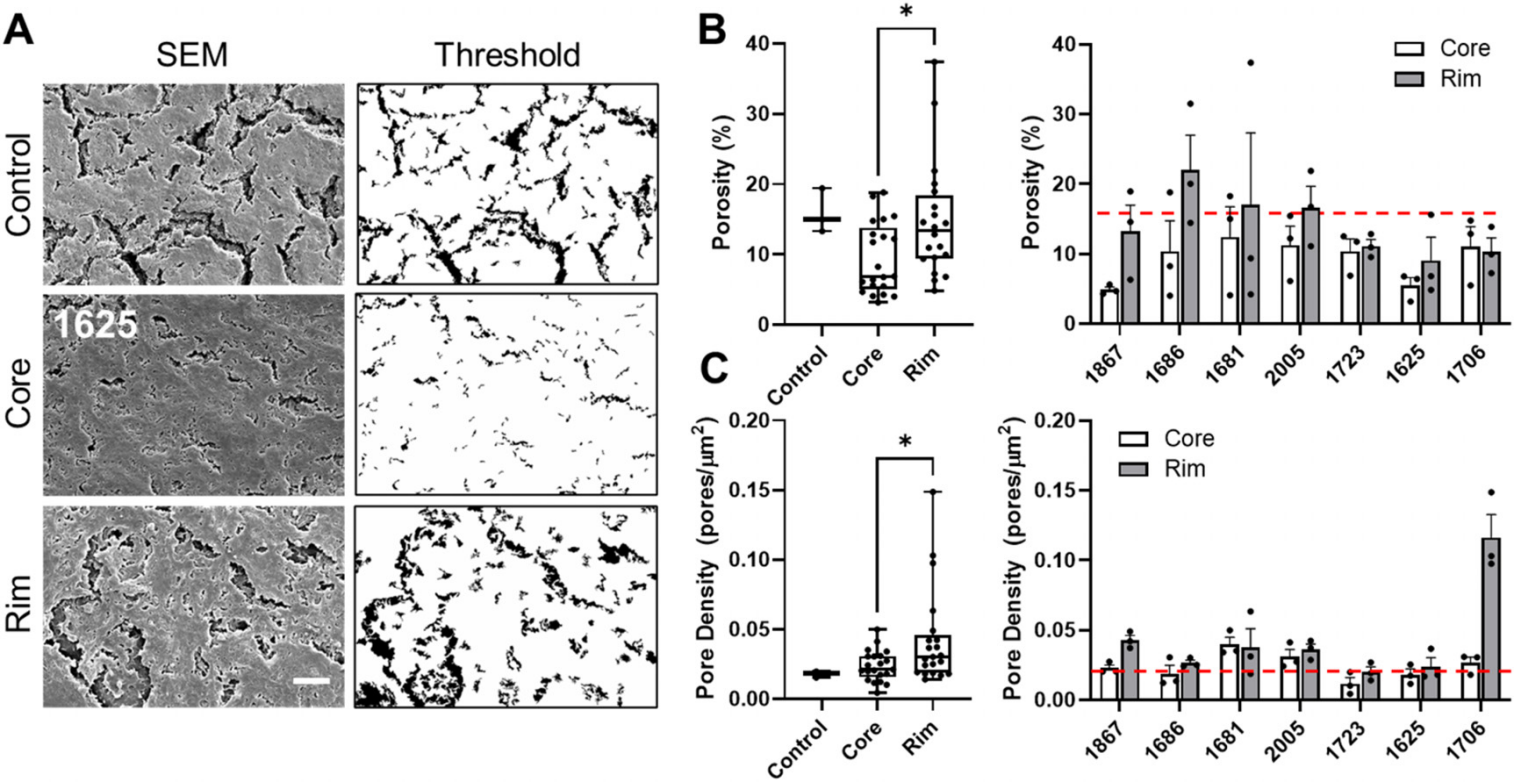
GBM core displays dense ultrastructure with decreased porosity and pore density; SEM analysis of three distinct regions in each core and rim sample (a) reveals decreased porosity within tumor cores when compared to tumor rim (p = 0.0384) and control samples (p = 0.2666). Patient-specific analysis show that porosity was higher in the rim for the majority of samples (b). Pore density, the number of pores per area, was also lower in the tumor core when compared to the rim (p = 0.0389) but similar to control levels (p = 0.914). Similarly, pore density was higher in the rim across patient samples. (c) Red dotted line indicates control measurements; statistical significance was determined via one-way ANOVA and Tukey's multiple comparisons test; ^*^ denotes p< 0.05, scalebar = 10 *μ*m.

To assess changes in tissue ultrastructure, we employed a strategy incorporating tissue decellularization and SEM imaging to quantify tissue ultrastructure, as described in Methods. We applied decellularization techniques to remove residual cells that may confound the analysis. GBM core displays dense ultrastructure with decreased porosity and pore density. Using standard porosity quantification methods as previously described, we found that tumor core tissue was less porous than rim and non-neoplastic control [Fig. 2(a)]. Tumor core tissue had a percent porosity that ranged from 4% to 12%, while rim tissue percent porosity ranged from 8% to 22% [Fig. 2(b)]. When all patient core and rim tissue porosity was averaged, tumor core tissue were significantly denser than tumor rim tissue. Similarly, tumor core exhibited lower pore density, defined as the number of pores per square micrometer [Fig. 2(c)]. The average pore density of tumor cores was also significantly lower than the tumor rim. Altogether, these findings support the AFM data and indicate that denser tissue is also mechanically stiffer.

### Glioblastoma core exhibits elevated hyaluronic acid and tenascin-C

**FIG. 3.**
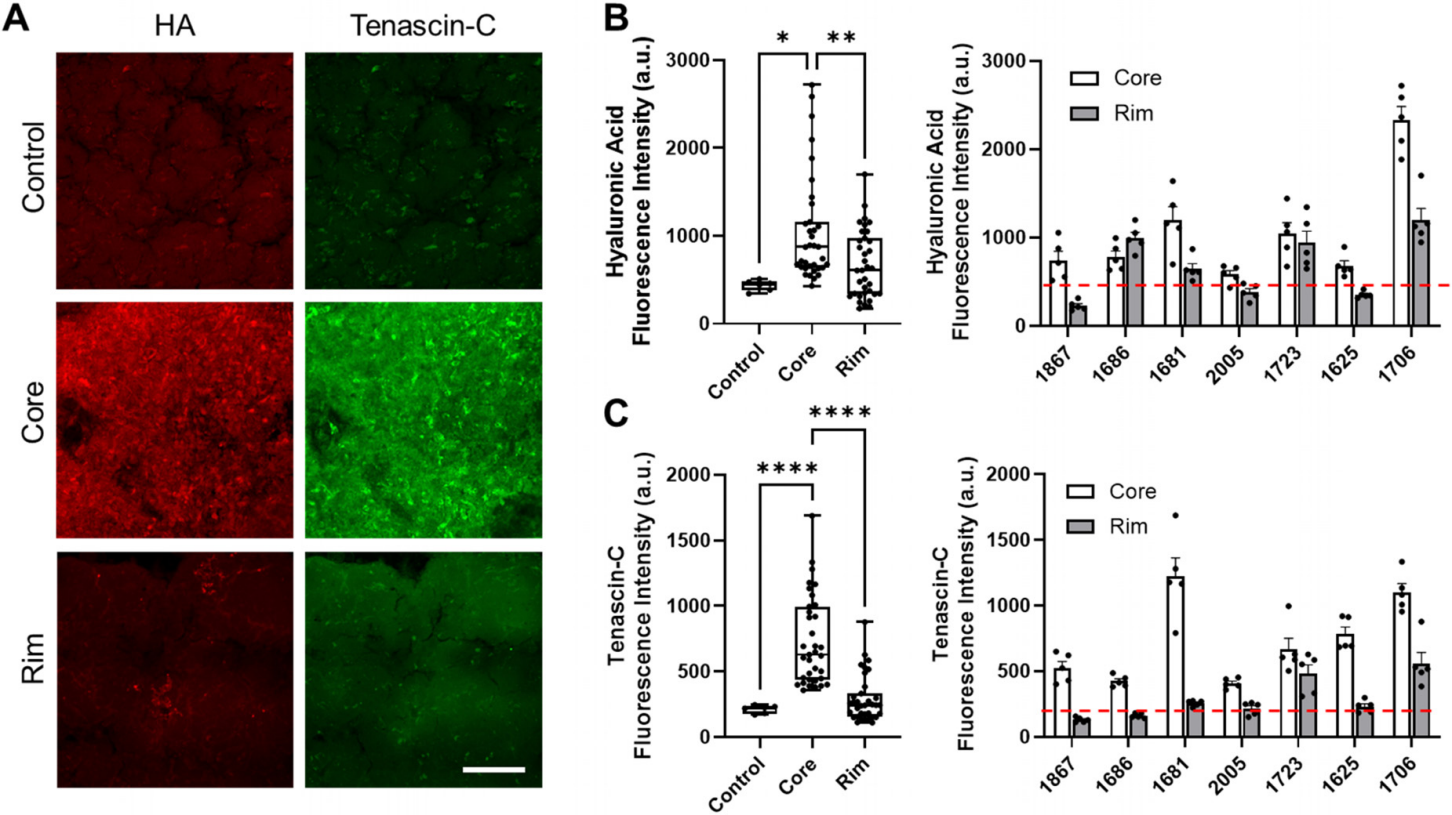
GBM core exhibits high hyaluronic acid and tenascin-C content immunostaining; immunostaining of HA and tenascin-C reveal that tumor core and rim exhibit elevated expression of both HA and tenascin-C, while the tumor rim exhibits a non-significant increase compared to the control tissue (a)–(c). Quantification of patient-specific changes show that tumor cores had elevated HA and tenascin-C when compared to the rim (b) and (c); red dotted line indicates control measurements; statistical significance was determined by one-way ANOVA and Tukey's multiple comparisons tests; ^*^ denotes p <0.05, ^**^ denotes p < 0.01, ^****^ denotes p <0.001; scalebar = 100 *μ*m.

To determine ECM differences between tumor core, rim, and non-neoplastic tissue, we conducted immunostaining on two parenchymal ECM proteins known to affect tissue organization and mechanics [Fig. 3(a)].^29^ The mean fluorescent intensity was taken at five distinct regions of interest across each patient sample. Using this semi-quantitative approach, we determined that both tumor core and rim had elevated levels of HA and tenascin-C when compared to non-neoplastic control tissue; however, only core tissue was significantly higher than the control [Figs. 3(b) and 3(c)]. Additionally, both patient-specific and cohort analysis showed that the prevalence of HA and tenascin-C was elevated in the core than the rim [Figs. 3(b) and 3(c)].

### Biophysical signatures of glioblastoma core and rim are correlated with prognosis

**FIG. 4.**
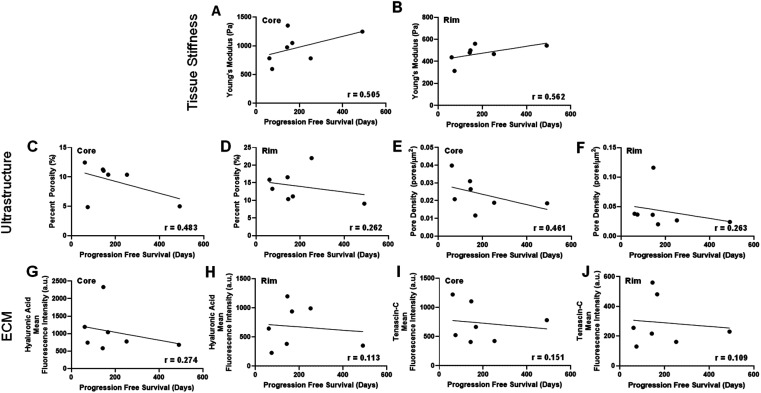
Biophysical signatures of GBM core and rim are correlated with survival. We observed moderate positive correlations (0.40 < r < 0.69) between stiffness and progression-free survival in both the core and rim (a) and (b). Moreover, we observed moderate negative correlations between porosity and pore density in the tumor core with a similar negative correlation in the tumor rim, which was determined to be a weak association (c)–(f). Finally, weak negative correlations (r < 0.40) were noted between ECM content and progression-free survival (G-J).

To consider the prognostic effect of the biophysical parameters in the tumor core and rim, we conducted linear regression analyses on the effect of stiffness, ultrastructure, and ECM composition on progression-free survival (PFS). We found that increased tissue stiffness had a positive moderate correlation with PFS in the core (r = 0.505) and in the rim (r = 0.562) [Figs. 4(a) and 4(b)]. Ultrastructural parameters show a negative moderate correlation in the core (porosity: r = 0.483 and pore density: r = 0.461) and a weak negative correlation in the rim (porosity: r = 0.262 and pore density: r = 0.263) [Figs. 4(c)–4(f)]. These data show that reduced porosity and pore density lead to increased PFS. Finally, analysis of correlations of ECM proteins and PFS revealed a weak negative association in both the core and the rim. The negative associations suggest that decreased ECM HA and tenascin-C content are associated with increased survival. In the core, HA and tenascin-C content had a weak negative correlation with PFS (HA: r = 0.274; tenascin-C: r = 0.151), and in the rim, HA and tenascin-C displayed similar trends (HA: r = 0.151, tenascin-C: r = 0.109) [Figs. 4(g)–4(j)].

### Increased pore density correlates with decreased PFS

To determine how specific GBM biophysical characteristics may influence PFS, patients were stratified into low (≤median; n = 4) and high (>median; n = 3) PFS groups. Mean core pore density was elevated in low PFS relative to high PFS patients (0.030 vs 0.016; p = 0.040; Table I). Similarly, pore density in rim was marginally elevated in low PFS patients (0.057 vs 0.024; p = 0.057; Table II). Both characteristics were negatively correlated with low PFS (p_corr_ = 0.012). This suggests that increased pore density in GBM may be associated with decreased survival.

**TABLE II.** Change in GBM biophysical characteristics between patients with low (≤median) or high (>median) progression-free survival (PFS). A negative correlation value is associated with decreasing PFS, while a positive value is associated with increasing PFS.

Biophysical Characteristic	Average low PFS	Average high PFS	P-value	Correlation value	Correlation P-value
Core modulus (mean)	926.665	1025.939	0.657	0.144	0.758
Porosity (core)	9.904	8.578	0.400	−0.433	0.332
Pore density (core)	0.030	0.016	0.040	−0.866	0.012
HA CORE (MFPA)	1212.263	832.463	0.413	−0.144	0.758
TC CORE (MFPA)	813.887	622.817	0.448	−0.144	0.758
Rim modulus (mean)	432.676	523.532	0.135	0.577	0.175
Porosity (rim)	14.021	14.071	0.991	−0.144	0.758
Pore density (rim)	0.057	0.024	0.057	−0.866	0.012
HA RIM (MFPA)	612.830	760.464	0.639	0.144	0.758
TC RIM (MFPA)	290.112	290.575	0.997	0.000	1.000

## DISCUSSION

The heterogenous nature of the TME underlies many of the challenges in treating GBM, and there is a critical need to better understand the components of the TME that promote disease evolution. Changes in the biophysical compartment of the TME have become recognized as significant regulators of progression, but their characteristics are still poorly described.^30,31^ Efforts to quantify these parameters is expected to generate insights for developing novel avenues for combating TME-dependent drivers of GBM progression. In this report, we find that tissue mechanics, ultrastructure, and ECM content vary significantly throughout the tumor core and rim in human patient-matched GBM samples. Furthermore, these parameters are correlated with patient outcome, suggesting that the biophysical characteristics of the GBM TME are relevant drivers in pathophysiology and should be considered in parallel to biomolecular signals.

Changes in tissue mechanics, ultrastructure, and ECM content have all been associated with tumor tissue in several tumor types; however, the spatial distribution of these changes and their impact on prognosis is poorly understood.^30,32^ Our findings support previous work, showing that tissue stiffness is associated with increased density and altered ECM remodeling.^33–35^ Correlation analysis of these three parameters show that increased tissue stiffness is strongly correlated with HA and tenascin-C, and that stiff tissues are denser and have less pores, although these correlations are much weaker (supplementary material, Fig. 1). In core/rim analysis, we find that the tumor core is much stiffer, denser, and rich in ECM when compared to the rim, providing evidence of heightened tissue remodeling at the core and a more porous and invasive margin at the rim.^36^ We show that stiff regions in the tumor are rich in HA and tenascin-C, consistent with elevated tenascin-C cross-linking with HA, which is known to stiffen the matrix and activate pro-malignant mechanosignaling.^29^ Our data provide a descriptive model showing that tumor cores possess a higher density of pro-malignant biophysical signals, including matrix stiffness and elevated ECM-cell signaling, presenting a pathological environment by which cells can adopt a malignant phenotype in the core and migrate toward a more permissive route of infiltration in the surrounding rim. These findings identify two tumor regions with different contributions to progression and, therefore, could serve as a basis for developing targeted therapies that are region-specific.

The analysis of correlations between biophysical signatures and progression-free survival in our cohort revealed interesting trends on the cumulative effect of stiffness, density, and ECM content. Surprisingly, our data show that increased stiffness in the core and rim moderately correlated with increased progression-free survival [Figs. 4(a) and 4(b)]. On the other hand, porosity and pore density were negatively correlated with survival in both the core and the rim [Fig. 4(c)–4(f)]. Similarly, in comparisons between low and high PFS groups, core pore density was significantly elevated in the low PFS group, suggesting that the number of pores may allow for more migration paths for infiltrating cells. Although increased stiffness is commonly associated with malignancy,^18^ our results are consistent with previous studies that show that tumor spheroids struggled to migrate in stiff and dense 3D matrices when compared to soft and porous gels.^28^ This highlights an important distinction between the effect of these parameters in 2D vs 3D and suggest that the combined effect of stiffness and porosity may influence 3D invasion and thus have prognostic effect.^24,37,38^ Correlation analysis of stiffness and ultrastructure in our patient cohort provides evidence that stiff tissue correlates with denser ultrastructure (supplementary material, Fig. 1). However, our results also show that several patient samples had stiff tumors and short progression-free survival, suggesting that the relationship between stiffness and ultrastructure is more complex and likely involves the presence of other TME components, including anatomical regions or perivascular niches.^39^ Our data also reveal a weak negative correlation between ECM content and progression-free survival, suggesting that patients with higher levels of HA and tenascin-C have worse prognosis [Figs. 4(g)–4(j)]. This finding supports observations that elevated ECM-receptor signaling, such as CD44 activation, can promote more aggressive and therapy resistant phenotypes in GBM and activate immune cells that facilitate disease progression.^27,40–43^

This is to our knowledge the first report on the heterogeneous biophysical characteristics of the aceullar human GBM TME in patient-matched samples. Limitations of this study include a small sample size, semi-quantitative approaches in image characterization, and a lack of omics-level analysis. Expanding the sample size and leveraging more quantitative tools will offer clearer insights and correlative trends than what is presented in this report. However, despite these limitations, the data show the broad mechanical, ultrastructural, and ECM changes in GBM and how the biophysical TME differs between patients. Additionally, our data reveal the integrative nature of biophysical parameters and identify an important role for pore density in patient survival. Larger cohorts should be studied in the future to identify trends/effects of patient demographic variables. Proteomic approaches that offer quantitative readouts of ECM levels would provide deeper insights. A larger dataset could also open analytical possibilities using mathematical modeling and machine learning. Importantly, identification of biophysical feature sets could be leveraged in the design of TME-targeted treatment options to combat GBM therapeutic resistance and improve patient outcome.

## METHODS

### Patient sample tissue and collection

**FIG. 5.**
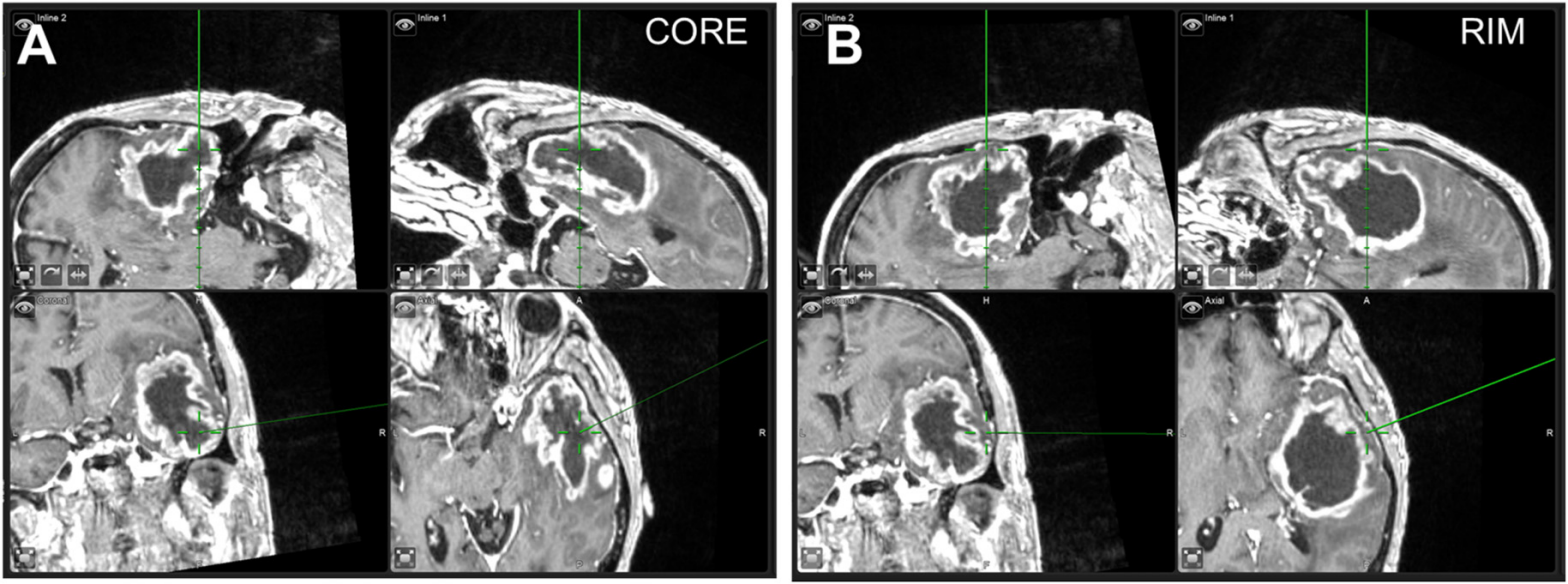
Intraoperative images from neuronavigation demonstrating representative biopsy sites at the crosshairs on the image. The top two images demonstrate trajectory/surgical views. The bottom two images demonstrate anatomical planar images in the coronal and axial planes. Image demonstrating core biopsy site (a) and rim biopsy site (b).

All specimens were collected following approval from the Internal Review Board protocol at the University of Louisville Hospital (IRB 20.0219) from patients with known or suspected brain tumors. As brain cancer affects both women and men, samples from both were collected. Samples were collected by the clinical team (seven patient-matched core and rim and one non-neoplastic tissue), blinded to the research analysis (Fig. 5). Patient information was de-identified by the clinical team before evaluation by the research team. Informed consent was obtained to participate in this study.

### Mechanical characterization via AFM

An Asylum MFP-3D-BioAFM (Oxford Instruments, Abingdon, UK) was used for all measurements. For sample preparation, OCT embedded brain samples were sectioned using a cryostat to 50 *μ*m thickness onto charged glass slides. All samples were analyzed in fluid with PBS without calcium and magnesium. MLCT-BIO cantilevers (nominal tip size ∼20 nm) (Bruker) were calibrated in Igor Pro to acquire the Defl InvOLS and the spring constant before testing. For data acquisition, a scan size of 20 × 20 *μ*m^2^ (max) was used with 16 × 16 pixel resolution yielding 256 data points per force map. For each force map, a ramp rate of 1 hz was used with an indentation depth of 3–5 *μ*m. A minimum of three force maps were acquired per sample, and an average mean stiffness was determined from the distribution for each set of patient samples. For calculation of the tissue modulus, a Poisson ratio of 0.5 was used, and the cantilever was approximated to a cone with a half angle of 36°. Adjacent sections were taken for ultrastructural and ECM analysis.

### Tissue decellularization

Tissue decellularization was conducted to visualize the ECM organization and ECM content of tissue sections without the interference of the presence of cells. A previously published decellularization protocol for brains was modified and used.^44^ OCT sections were thawed at room temperature for 60 min. A hydrophobic pen was used to outline the tissue and support the incubation of decellularization reagents. Tissues were rinsed in PBS and de-ionized water before incubation in 0.1% sodium deoxycholate (SD) for 15 min at 37 °C. Tissues were then rinsed in PBS and incubated in DNAse I solution for 35 min at 37 °C. After removal of DNAse I, tissues were rinsed three times in PBS for 5 min before SEM and immunostaining.

### SEM preparation and ultrastructural quantification

For SEM imaging, decellularized tissues were process as previously described.^45^ Briefly, samples^45^ were fixed in 2.5% glutaraldehyde in 0.1M sodium cacodylate (pH 7.4). Samples were then rinsed in 0.1M PBS three times for five minutes each at room temperature. Tissues were incubated in 1% osmium tetroxide for 30 min and then rinsed with de-ionized water three times for 10 min each. Finally, samples were dehydrated sequentially with 50%, 70%, and 95% EtOH for 10 min each. Prior to imaging, a Cressington Vacuum Coating System for Au and Au/Pd (Cressington Scientific, Watford, UK) was used to sputter coat fixed tissue for 30 s or to approximately 7 nm thickness. Images of processed samples were obtained via Apreo C LoVac FESEM (Thermo Scientific, Waltham, MA) at a working distance between 11 and 15 mm, with a high voltage of 5.00 kV, using the Everhart–Thornley Detector (ETD), and the magnification ranged from 200× to 2000× based on the size of the sample, with a probed depth of ∼1 *μ*m.

SEM images were used to calculate porosity, average pore size (*μ*m^2^), and pore density (pores/*μ*m^2^) via ImageJ (NIH, Bethesda, MD). Using porosity quantification strategies as previously described, thresholding techniques were used to identify pores in each sample.^46^ Porosity was determined as the total porous area/total image area; the average pore size was determined by the total porous area/total number of pores; the pore density was determined by calculating the total number of pores/total image area.

### Immunofluorescent staining and quantification

Samples were fixed in 3.7% paraformaldehyde, washed with PBS, and then blocked for one hour. After blocking, samples were incubated with primary antibodies against hyaluronic acid (HA) and tenascin-C at 1:200 dilution for 3 h at room temperature. After rinsing with PBS, secondary antibodies at a 1:1000 dilution were added for one hour. Samples were then mounted with anti-fade mounting media overnight before imaging using a Nikon A1 Confocal Microscope (Nikon Instruments, Melville, NY). Fluorescence area and intensity was calculated as previously described.^47^ Briefly, five regions of interest were selected across each sample, and the corrected total fluorescence was calculated for each using the equation CTF = Integrated Density—Total Fluorescent Area/Background Fluorescence via ImageJ.

### Statistical analysis

The data are reported as the mean of all replicates, and error is given as standard error of the mean. For correlation analysis, the Spearman's coefficient was generated through linear regression analysis via GraphPad Prism 9 and a value of 0.40–0.69 and 0.70–0.89 was deemed a moderate and a strong correlation, respectively, as previously described.^48^ For comparison of low and high PFS groups, statistics were performed using R 4.2.2. P-values were computed using T-test and Wilcoxon Rank Sum tests for parametric and non-parametric data, respectively. Correlations were computed from *cor.test* using Spearman correlations. Normality was determined using Shapiro–Wilks test (p ≤ 0.05). GraphPad Prism 9 software was also used to create figures, and statistical significance between sample groups was determined by one-way ANOVA and Tukey comparison tests unless otherwise noted. Details of comparisons and replicates are provided in the appropriate figure legend.

## SUPPLEMENTARY MATERIAL

See the supplementary material for additional figures on correlations between biophysical parameters.

## Data Availability

The data that support the findings of this study are available from the corresponding author upon reasonable request.
